# Determination of biological activity of gonadotropins hCG and FSH by Förster resonance energy transfer based biosensors

**DOI:** 10.1038/srep42219

**Published:** 2017-02-09

**Authors:** Olga Mazina, Anni Allikalt, Juha S. Tapanainen, Andres Salumets, Ago Rinken

**Affiliations:** 1 University of Tartu, Institute of Chemistry, Tartu, Estonia; 2 Competence Centre on Health Technologies, Tartu, Estonia; 3 Department of Obstetrics and Gynecology, University of Helsinki and Helsinki University Hospital, Helsinki, Finland; 4 Department of Obstetrics and Gynaecology, University of Tartu, Tartu, Estonia; 5 Institute of Biomedicine and Translational Medicine, University of Tartu, Tartu, Estonia

## Abstract

Determination of biological activity of gonadotropin hormones is essential in reproductive medicine and pharmaceutical manufacturing of the hormonal preparations. The aim of the study was to adopt a G-protein coupled receptor (GPCR)-mediated signal transduction pathway based assay for quantification of biological activity of gonadotropins. We focussed on studying human chorionic gonadotropin (hCG) and follicle-stimulating hormone (FSH), as these hormones are widely used in clinical practice. Receptor-specific changes in cellular cyclic adenosine monophosphate (cAMP, second messenger in GPCR signalling) were monitored by a Förster resonance energy transfer (FRET) biosensor protein ^T^Epac^VV^ in living cells upon activation of the relevant gonadotropin receptor. The BacMam gene delivery system was used for biosensor protein expression in target cells. In the developed assay only biologically active hormones initiated GPCR-mediated cellular signalling. High assay sensitivities were achieved for detection of hCG (limit of detection, LOD: 5 pM) and FSH (LOD: 100 pM). Even the small-scale conformational changes caused by thermal inactivation and reducing the biological activity of the hormones were registered. In conclusion, the proposed assay is suitable for quantification of biological activity of gonadotropins and is a good alternative to antibody- and animal-testing-based assays used in pharmaceutical industry and clinical research.

Gonadotropin medications are widely used in controlled ovarian stimulation and induction of ovulation as key components of infertility treatment. A number of gonadotropin preparations are available, based on the naturally occurring gonadotropins: follicle-stimulating hormone (FSH), luteinizing hormone (LH), and human chorionic gonadotropin (hCG).

Gonadotropins are glycoprotein hormones that regulate normal growth, sexual development, and reproductive function. These are large, up to 40 kDa proteins, which are synthesized and secreted by the gonadotropic cells of the anterior pituitary gland (LH and FSH) and by the syncytiotrophoblasts in the placenta (hCG). These hormones may vary in the level of glycosylation depending on myriad of physiological co-factors. Upon binding to FSH receptor (FSHR), a G-protein coupled receptor (GPCR), FSH regulates the development, growth, pubertal maturation, and reproductive processes, like maturation of germ cells in both women and men. LH and hCG bind to a shared GPCR (LH/CG receptor, LHCGR) and regulate mechanisms involved in ovulation, early pregnancy and placental function, respectively, in females, while in men, LH is involved in spermatogenesis and testosterone production^1^.

FSH, LH and hCG are heterodimeric proteins that consist of non-covalently linked α- and β-subunits. The gonadotropins share the α-subunit structure, but are each bound to a unique β-subunit resulting in differences in their physiological roles and signalling^1^. Upon binding to their receptor, gonadotropins activate the Gs-coupled signalling pathway resulting in stimulation of a family of cellular enzymes, adenylate cyclases. These enzymes catalyse production of the second messenger molecule cyclic adenosine monophosphate (cAMP) from cellular pool of ATP. cAMP in turn acts via its many effector proteins to result in a cellular response to receptor activation^2^,^3^.

There are a number of gonadotropin preparations commercially available or in development, including both urinary-derived hormones and those produced through recombinant DNA techniques^4^,^5^. Moreover, differences in the glycosylation patterns of hCG and FSH give rise to a number of naturally occurring isoforms that may differ functionally. The relative concentrations of these isoforms vary over the course of the menstrual cycle, pregnancy and lifetime, indicating that differences in glycosylation may have physiologic relevance^6^.

Recently, many reports have been published that state gonadotropins as biomarkers for cancer and other diseases. The beta subunit of hCG has been shown to be expressed at low concentrations by many cancers and it might serve as a prognostic marker^7^,^8^,^9^. Also, FSH receptor has been shown to be expressed in tumour blood vessels of several cancers^10^. Thus, FSHR expression in endothelial malignant cells could have a predictive impact on disease progression, especially in relation to therapies targeting the tumor vasculature^11^.

Over the last 50 years many assays have been developed for gonadotropin detection relying on *in vivo* physiological changes caused by gonadotropins on cells or animals, e.g. measurements of estradiol production in rat Sertoli cell culture after addition of FSH, testosterone production by mouse Leydig cells after LH or hCG stimulation or the linear relationship between administered gonadotropins and ovarian weight measured in rats post-mortem^12^. In addition, many sensitive and very specific immunological assays have been introduced based on interactions of hormones with their specific antibodies^9^,^13^,^14^. However, till date there is no real-time assay available to measure the concentrations of only the biologically active gonadotropins.

In the current study we have implemented the gonadotropin receptor-specific assay system, where the bound active hormone is detected by cellular production of cAMP as a result of the receptor activation. The changes in cAMP levels were detected using a Förster resonance energy transfer (FRET) based biosensor protein^15^ ^T^Epac^VV^. The biosensor consists of a part of a cAMP binding protein (Epac1) fused between a bright fluorescent protein mTurquoise and a tandem acceptor consisting of two Venus proteins. After excitation of the donor fluorescent protein mTurquoise at 430 nm apart from light emission upon relaxation, some of the energy is transferred and used to excite the acceptor fluorescent protein Venus, due to the overlap of donor emission and acceptor excitation spectra. Cellular cAMP, after binding to the biosensor, distorts the molecular proximity and orientation of donor and acceptor fluorophores and FRET efficiency drops. By detecting fluorescence emission intensities of the biosensor at two wavelengths simultaneously (480 nm for donor emission and 530 nm for acceptor emission), an intensity ratio is determined prior to and after cell stimulation with the hormone. The change in emission intensity ratio is proportional to the change in cAMP concentration.

Recently, generation of an alternative cAMP sensor cloned into the BacMam system for cAMP detection was reported^16^. This “cAMP Difference Detector *in situ* (cADDis)” sensor uses only one circularly permuted green fluorescent protein, which is incorporated in the hinge region of Epac protein and yields a change in fluorescence emission signal at a single wavelength. This is practical if the dual simultaneous emission instrumentation is not available in the laboratory. However since FRET based sensors like ^T^Epac^VV^ report changes in signal at two separate channels, the calculated intensity ratio adds to the robustness of data analysis and serves as an internal control with no need to correct for photobleaching.

In the current study we show and compare two simple ^T^Epac^VV^ biosensor-based assays, which are specific to FSHR and LHCGR receptor activation in living cells, demonstrating that these sensors can be used for quantification of the biological activity of gonadotropins.

## Materials and Methods

**Figure 1:**
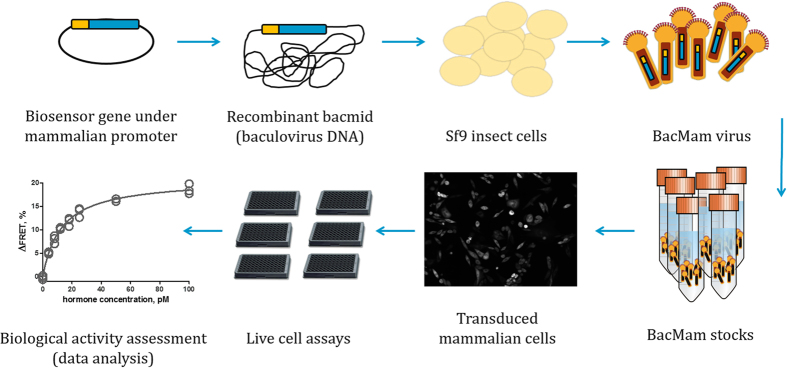
Generation of the BacMam delivered cAMP detection-based functional assay system for quantification of biologically active gonadotropins using living cells.

The baculovirus construct carrying the recombinant gene encoding for the cAMP biosensor was constructed as previously described^17^,^18^. In brief: The pcDNA3.1+ expression vector with the ^T^Epac^VV^ gene^15^ was cloned into the pFastBac™1 vector. The polyhedrin promoter from the pFastBac™1 vector was replaced with the powerful mammalian cytomegalovirus promoter. The resulting pFastBac-^T^Epac^VV^ construct was used to produce bacmid DNA (recombinant Sf9 cell genome), that was transfected into *Spodoptera frugiperda* Sf9 insect cells to obtain the BacMam virus. The virus was amplified and the stocks stored at −80 °C. The viral titers were determined experimentally by a cell size-based assay^17^ using Sf9 cells. For functional experiments the mammalian cells were treated with the BacMam virus at multiplicity of infection (MOI) between 100 and 300 infectious viral particles per mammalian cell. The general principle of the used BacMam technology is shown in (Fig. 1). pFastBac™1 vector and Sf9 cells were purchased from Invitrogen Life Technologies.

The Sf9 insect cells were cultured in suspension in EX-CELL 420 medium (Sigma-Aldrich) at 27 °C in a non-humidified incubator without supplements of serum or antibiotics. The mammalian cell lines (COS-7 cells expressing recombinant LH/CG receptor^19^,^20^ and KGN cells expressing the endogenous human FSH receptor^21^) were grown as adherent monolayers at 37 °C and 5% CO_2_ in a humidified incubator in Dulbecco’s Modified Eagle’s Medium and Ham’s F-12 nutrient mixture (DMEM/Ham’s F-12) growth medium supplemented with 10% fetal bovine serum, 100 U/ml penicillin and 0.1 mg/ml streptomycin. All mammalian cell culture media and supplements were from PAA Laboratories.

The assay to measure GPCR activation in living cells was performed as described previously^17^,^18^. In brief: mammalian cells were treated with viral stock (MOI: 100–300) in 4 ml of growth medium for 3–4 h at 30 °C in a humidified CO_2_ incubator. After preincubation with the virus, the medium was aspirated and replaced with 10 ml of fresh growth medium containing 10 mM sodium butyrate (Sigma-Aldrich) for enhanced protein expression. The transduced cells were cultured for further 21 h at 30 °C for recombinant protein production. On the day of the assay the cells were seeded on the black 96-well clear-bottom cell culture plate (Corning Life Sciences) in phosphate-buffered saline 1–2 h prior to the experiment, with assay volume of 100 μl. The fluorescence intensities were registered prior and after addition of the hormones using a Synergy™ NEO HTS Multi-Mode Microplate Reader (BioTek Instruments, Inc.) with filter-based detection at excitation wavelength of 420/50 nm and simultaneous dual emission at 485/20 and 540/25 nm. The change in the Förster resonance energy transfer (ΔFRET) was calculated using the following equation: 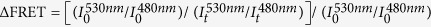, where 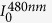
and 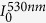 refer to the measured intensities of fluorescence emission for the donor and the acceptor fluorophores before addition of the hormone and 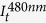 and 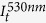 refer to the measured intensities at time point *t* after addition of the hormone. The experiments were performed at least on four independent assay days in triplicate. The limit of detection (LOD) was 3.3*σ/S, and the limit of quantification (LOQ) was 10*σ/S, where S is the slope of the calibration curve of the hormone and σ is the standard deviation of the blank.

For enzyme-linked immunosorbent assay (ELISA) and electrochemiluminescence immunoassay (ECLIA) samples of recombinant hormones were prepared in milli-Q water and divided into three. The first aliquot was heat-treated in a water-bath at 65 °C and the second at 95 °C for 30 minutes. The third sample was kept at room temperature as the untreated control. The hormones’ concentration was determined using ECLIA by Roche (performed in the United Laboratory of Tartu University Hospital) either for hCG standardized against the 4th International Standard for chorionic gonadotropin from the National Institute for Biological Standards and Control (NIBSC) code 75/589 or for human FSH against the 2nd International Standard NIBSC 78/549. All samples were analysed in duplicate on two independent assay days. hCG concentration was also determined by ELISA kit (ab100533, Lot: GR217510-1) purchased from Abcam (Cambridge, UK). The experiments were performed according to manufacturer’s manual in duplicate on two independent assay days.

The hormones used in this study were purchased from Merck Serono Europe limited. The initial concentration of the human recombinant chorionic gonadotropin (hCG, Ovitrelle) was 5,780 IU/ml = 500 μg/ml and of the human recombinant follitropin alfa (FSH, Gonal-F) 600 IU/ml = 44 μg/ml.

## Results and Discussion

We have previously shown that the cAMP biosensor ^T^Epac^VV^ is a reliable tool to follow the activation of different receptors, from low-molecular weight ligands like dopamine and adrenaline^22^ towards high-molecular-weight glycoproteins^18^. These studies demonstrated that the measured signal is specific to the ligands and receptors used. However, for high-molecular weight ligands, like glycoproteins FSH and hCG, the key question is whether cAMP biosensor is able to discriminate between the biologically active and inactive forms of the protein. This led us to set the general objective for the next study − to check the possibility of using our biosensor system for quantification of biological activity of different hormones. The focus was put on gonadotropins, as these hormones are clinically widely used and no direct and fast methods existed for measuring their biological activity. For these purposes, we selected two cell lines, COS-7 cells expressing recombinant LH/CG receptor and KGN cells expressing the endogenous human FSH receptor, which both have been shown to have good receptor level and fully functional signal transduction system^19^,^20^,^21^,^23^.

These receptors are coupled with the Gs pathway and therefore their activation can be measured with the ^T^Epac^VV^ cAMP biosensor^15^,^17^. However, for quantitative measurements, reproducible transfection of the sensor has to be achieved. For that we used the BacMam technology^24^, by transferring the gene encoding for the biosensor into target cells with recombinant baculoviruses carrying the gene under a strong cytomegalovirus promoter (Fig. 1). The transgene with the mammalian promoter is cloned into the baculovirus genome (bacmid), which is transfected into the host cells (Sf9) for virus production and amplification. The Sf9 cells do not produce the biosensor protein due to lack of an insect promoter in front of the transgene, and are thus used only in virus producing step. The virus is collected from Sf9 cells’ supernatant and stored at −90 °C. Depending on the mammalian cell-line of choice, multiplicity of infection of about 100–300 of virus particles per cell is used to obtain sufficient and homogeneous expression of the biosensor^17^. There are no limits to the amount of cells to be transduced with the virus, making this approach suitable for assay platforms where large amounts of samples are needed and higher numbers of biosensor expressing cells are desired. The level of expression of the biosensor can be adjusted by the viral dose and followed by the level of fluorescence intensity resulting from excitation on the day of the assay. Here, the developed biosensor expression system was successfully applied in both cell lines used, supporting our previous evidence that this technology is compatible with several different mammalian cell lines and GPCRs of interest^17^.

**Figure 2:**
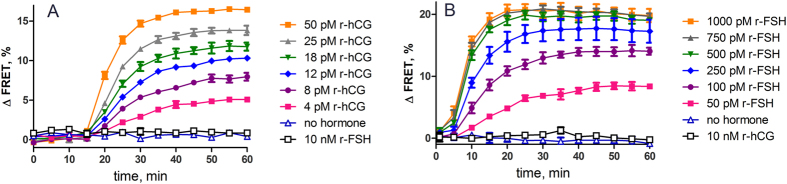
Time dependent change of FRET signal in response to different concentrations of hCG and FSH. COS7 cells expressing recombinant LH/CG receptor **(A)** and KGN cells expressing the endogenous FSH receptor **(B)** were transduced with BacMam-^T^Epac^VV^ virus and incubated with serial concentrations of recombinant hCG **(A)** and FSH **(B)** for indicated time. The graphs show data points with connecting lines from a representative experiment performed in triplicate (n = 4).

Stimulation of the cells with either of the hormones caused concentration and time-dependent activation of the corresponding GPCR and was detectable by the changes in the biosensor signal (Fig. 2). The time required to activate the cellular cascade was different for the two cell lines expressing the particular gonadotropin receptor. A clear lag-phase of about 15 minutes for hCG and 5 min for FSH was needed to activate the receptor-mediated signalling, but in both cases the signal stabilised within one hour after stimulation for all of the measured hormone concentrations. Hence, for later quantitative experiments signal acquisition at 60 minutes after ligand addition was used. Moreover, FSH at concentrations up to 10 nM caused no detectable activation in COS7-LHCGR cells indicating the specificity of the assay, as full receptor activation was achieved already at 50 pM hCG concentration (Fig. 2A). Similar results of no receptor activation were obtained for FSHR in KGN cells with 10 nM hCG (Fig. 2B). The slower response found for large hormones, like hCG and FSH may be connected with their slower binding to the receptors. Low-molecular weight hormones as adrenaline or dopamine and peptides like melanocortin receptor agonists, which bind fast to their receptors, reveal a stable cAMP signal within 10 minutes after addition of the ligand^22^,^25^. Relatively slow kinetics of gonadotropin hormones’ binding to their receptors have also been shown with ^125^I-labelled radioligand binding experiments to cell membrane preparations^26^,^27^,^28^,^29^ and have been explained by hindrance due to steric factors and ligand binding interaction at the binding sites.

**Figure 3:**
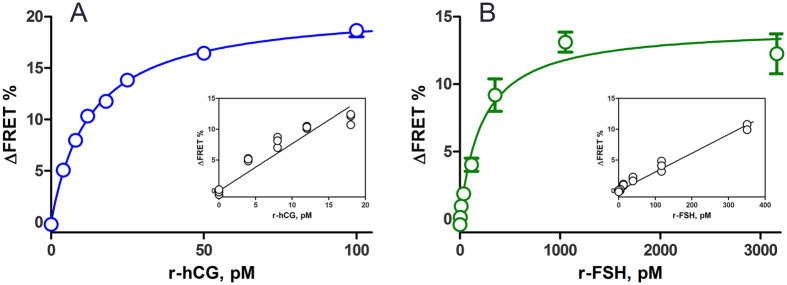
Change of FRET signal at different concentrations of hCG and FSH. COS7 cells with LH/CG receptor **(A)** and KGN cells with FSH receptor **(B)**, transduced with BacMam-^T^Epac^VV^ virus were incubated with different concentrations of recombinant hCG **(A)** and FSH **(B)** for 60 min. The graphs show data points from a representative experiment performed in triplicate (n = 4). The insets show the linear area of the response that was used for estimation of the limit of detection and quantification.

The concentration-dependent change in the measured FRET signal proved the suitability of the assay for quantification of hormone concentration. The dependencies between the hormone amounts and the sensors’ responses were not strictly linear and data could be fitted to one-site binding hyperbola and used as calibration curves (Fig. 3). The linear parts of these curves allowed estimating the detection limits of the assays (Fig. 3 insets). As can be seen from the figures, the assays of gonadotropin quantification are very sensitive, having LOD ~ 5 pM for recombinant hCG and LOD ~ 100 pM for recombinant FSH. Still, these LOD values report the limits of these particular cellular systems, as difference in receptor expression levels (and therefore the pool of spare receptors) could influence the detection limits of the assay. If our technology for determination of hCG and FSH will be adopted in other cellular systems; the obtained LOD values could vary and should be determined in the process of assay setup. These differences, however, are not expected to be large compared to the scale determined in this study if genetically modified receptors are not used.

In case of such a high achieved sensitivity of the developed assay, the adsorption of the hormone onto the surface of test tubes or pipette tips may lead to under- or overestimation of the measured response. For testing of this kind of a potential problem, we have used masking protein – bovine serum albumin (0.1 mg/mL) and mild detergent pluronic acid (0.1%) to decrease possible non-specific binding. However, none of these reagents had significant influence on the concentration dependence of cellular signalling detected (data not shown). This indicated that it is not necessary to use these reagents to stabilize hormones in solution. Furthermore, in order to check the loss of the hormone during the dilutions, we performed serial pipetting assay. In these experiments 1 mL solutions of 50 pM hCG and 500 pM FSH were prepared and serially transferred to the next tubes leaving 100 μl of the solution behind. Thereafter eight of consequent left-behind samples were assayed. No significant losses of hormones during these transfers were detected, as all of the measured samples caused similar change of the biosensor signal (data not shown). Hence we can conclude the robustness of the method in our hands, but the risk of non-specific ligand binding has to be assessed before the assay will be implemented with new materials.

**Figure 4:**
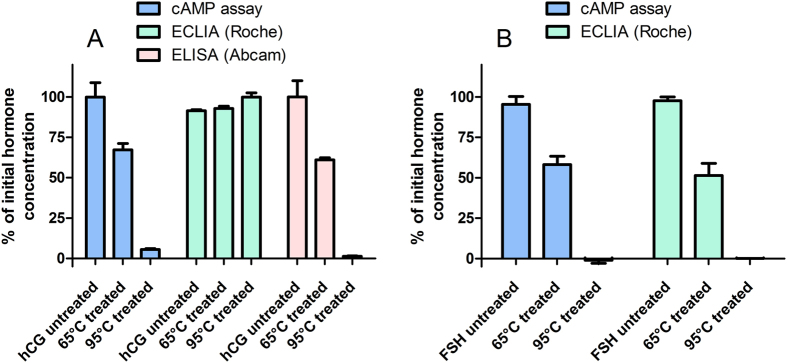
Influence of heat treatment on activities of recombinant hCG (**A**) and FSH (**B**) as measured by cAMP biosensor assay, electrochemiluminescence immunoassay (ECLIA) and enzyme-linked immunosorbent assay (ELISA). The hormone preparations were incubated at room temperature (untreated), at 65 °C or at 95 °C for 30 min and then their activities were measured. The concentration of hCG was 30 pM (cAMP assay) or 150 pM (ECLIA and ELISA) and the concentration of FSH was 300 pM. Data were normalized against activities of hormones kept at room temperature. Data are presented as mean ± SEM of 2 independent experiments carried out in triplicates.

Because the assay relies on the measurement of cellular response after receptor activation by the hormone, only the level of biologically active hormone is detected rather than the total hormone concentration in the sample. To test this, hCG and FSH samples were heated up to 65 °C and 95 °C and kept at these temperatures for 30 minutes. After the heat treatments the hormones’ abilities to generate signal in corresponding cells were significantly decreased. The treatment at 65 °C caused about 40% loss in active hormone concentration, which means that more than one third of the hormones had been inactivated and lost the ability to activate receptor-dependent signal transduction pathway (Fig. 4). While the treatment at 95 °C led to full deactivation of both hormones. These experiments proved that the hormones’ structures are temperature sensitive and only the active conformation of gonadotropins induces the measurable response.

As not only heating, but also freezing may affect protein’s conformation and activity, we studied its influence on the gonadotropins’ activity. The hCG and FSH samples were frozen and stored at −20 °C for at least one month. No significant changes in their activity were found even though they passed several thawing and freezing cycles (data not shown). This means, that although these gonadotropins are temperature sensitive, the freezing and thawing do not significantly hamper their biological activity.

Similar thermostability experiments were performed also with commercially available assay systems. We used ELISA assay kits from Abcam (ab100533), which have been reported to be generated for active hormones and ECLIA assays by Roche, routinely used in clinical laboratories (Fig. 4). In contrast to our assay, the ECLIA assay for detection of hCG did not distinguish the heat-inactivated hormone from the hormone kept at room temperature, showing that it cannot be used to distinguish active hormone from the denaturated one.

It is known that antibodies can be generated against different protein epitopes and only some of them are recognizing the epitopes responsible for the functional activity of the protein. Therefore a wide range of antibodies available for diagnostic kits and created against different epitopes may result in a broad range of testing results, which usually do not correlate with the functional characteristics of the protein^14^. This was also seen in above described experiments, where one assay system also identified the fully inactivated proteins, like ECLIA assay for hCG. Indeed, as immunological methods rely on antibody-antigen interaction, they do not provide information about the biological activity of the antigen. Therefore the direct measurement of the hormone’s biological activity is needed and the receptor-dependent cAMP-assay developed in the current study fully corresponds to these requirements. In fact, in our assay the signal is generated only when the biologically active and intact hormone is bound to its receptor, e.g. to its natural target. Moreover, in the ligand-receptor interaction and cellular response the whole hormone is involved, which is different from the locally restricted recognition of antigen by its antibody.

The developed assay can also be useful in analysing the biological activity of various gonadotropin isoforms in different physiological conditions, e.g. thorough the entire menstrual cycle or in pathogenesis of different reproductive diseases. If the functional assay reporting the bioactive hormone level (B) is used in parallel with assays reporting immunoreactive (I) concentrations, the B:I ratios can be determined for various samples containing hCG, LH or FSH. The B:I ratio has been shown to be potentially important parameter to characterize patient’s reproductive health and pregnancy outcome^12^,^30^,^31^. Thus, investigation of hormonal B:I changes over time could be of interest in patients with various reproductive illnesses, and the assay system developed in the current study would greatly facilitate this type of clinical studies in the future.

The requirement to determine the biological activity of a hormone or any other drug is the policy of European and US Pharmacopoeias. These traditional tests are usually complex assays, which require the use of living animals and thus represent uncomfortable, unwarranted and unethical way for analysis of the biological activity of different compounds. For example, the *in vivo* Steelman and Pohley bioassay for FSH is based on the female rats being sensitive to exogenous FSH and showing the linear relationship between administered FSH and ovarian weight^32^. Given the general trend of replacing the animal testing with accurate *in vitro* assays, the development of cAMP cell-based assay for active gonadotropin detection should be more than welcomed by pharmaceutical industry and clinical research community.

Indeed, in the current study we proposed an assay system, which can be relatively easily implemented and used by laboratories with bioanalytical capabilities. Importantly, no animals need to be sacrificed for experimental work, which is more in-line with the recommendations to avoid using animal testing in pharmaceutical industry. Furthermore, the biosimilar gonadotropin preparations used in assisted reproduction are emerging and are expected to be biologically and clinically ‘non inferior’ to the original products^5^. The cAMP assay can provide a tool to compare the new products with the originals. Also, the assay could be of help as a reference method to compare and characterize the specificity of antibodies against gonadotropins used in various immunoassays.

In conclusion, the implemented assay for detection of biological activities of hCG and FSH, relying on hormone receptor activation and the subsequent cAMP production, offers a valuable alternative for evaluation of different hormone preparations. We have demonstrated high sensitivity and reproducibility of the assay for hCG and FSH testing, which could potentially be used in clinical research and pharmaceutical industry.

## Additional Information

**How to cite this article**: Mazina, O. *et al*. Determination of biological activity of gonadotropins hCG and FSH by Förster resonance energy transfer based biosensors. *Sci. Rep.* **7**, 42219; doi: 10.1038/srep42219 (2017).

**Publisher's note:** Springer Nature remains neutral with regard to jurisdictional claims in published maps and institutional affiliations.
